# Role of Accumulated Calcium in Alleviating Aluminum Injury in Wheat Plants

**DOI:** 10.1155/2014/457187

**Published:** 2014-02-20

**Authors:** M. Alamgir Hossain, M. Ashrafuzzaman, A. K. M. Zakir Hossain, Mohd. Razi Ismail, H. Koyama

**Affiliations:** ^1^Department of Crop Botany, Bangladesh Agricultural University, Mymensingh 2202, Bangladesh; ^2^Institute of Tropical Agriculture, Universiti Putra Malaysia (UPM), 43400 Serdang, Selangor, Malaysia; ^3^Laboratory of Cell Technology, Gifu University, Japan

## Abstract

Aluminum (Al) sensitive wheat cultivar kalyansona was grown for 14 d in a range of Ca solution (125, 625, and 2500 **μ**M) plus other nutrients without Al. At 14 d after Ca treatment, half of these plants were harvested (H1), and the rest of the plants were exposed to 100 **μ**M Al for additional 6 d and harvested (H2). Severe Al injury was found only in the plants with the lowest supply of Ca before Al treatment. Aluminum concentration in the apoplastic fluid was very high at 125 **μ**M Ca probably because the plasma membrane of some of the cells was destroyed due to the attack of 100 **μ**M Al. Aluminum content in roots decreased with increasing supply of Ca before Al treatment. Calcium content decreased drastically at harvest (H2) in the plants with 100 **μ**M Al. Under Al stress conditions, the plant responded to Al in different ways due to not only the different Ca supply but also the variation of Ca content in the plant tissues. Actually, the plants having the largest Ca content in the roots before Al treatment can receive less Al injury during Al treatment. To substantiate this idea, a companion study was conducted to investigate the effects of 2500 **μ**M Ca supply during, before, and after 100 **μ**M Al treatment on root growth. The results indicated clearly that exogenous Ca supply before Al treatment is able to alleviate Al injury but less effective than Ca supply during Al treatment.

## 1. Introduction

The effects of Aluminum on the uptake and accumulation of divalent cations such as Ca and Mg have been extensively studied [1, 2]. It has been accepted that Al reduces accumulation of divalent cations, especially by displacing Ca from the cell wall as well as plasma membrane and that higher levels of Ca in the solution can alleviate deleterious Al effects [3–6]. Recently, we found that Al displaced some part of Ca in cell walls of wheat roots when they were exposed to Al [7] and additional Mg was less effective in alleviating Al injury when exogenous Ca supply was high [8]. The ability to prevent displacement of Ca in the root apoplast by Al has been suggested as one of the mechanisms determining Al tolerance in plants [4]. So, Al tolerance has been considered to be associated with the ability of absorbing and utilizing Ca when Al is present. There have been conflicting reports regarding whether Ca influx into plant cells is inhibited by Al. The inhibition of Ca uptake in roots by Al has long been considered a possible cause of toxicity [9], but later reports suggested that the inhibition of root growth is not caused by the reduction of Ca uptake [10, 11].

Considerable evidence suggests that the phytotoxic effects of Al on roots can be partially or completely overcome by increasing the concentration of Ca in the culture solution [4, 12, 13]. This phenomenon is not solely due to changes in external Al activity but also related to Ca nutrition. From reviewing the above-mentioned reports, it is clear that increased supply of Ca in the culture solution alleviated Al toxicity by improving Ca status of the plants. Therefore, the status of Ca of a plant is an important factor to explain the alleviation mechanism/strategies of Al toxicity. Calcium and Al were applied simultaneously in the culture solution in most of the experiments studying for Al-Ca interactions. But literature is very scarce for studying the interactions by applying Ca before Al treatment. Therefore, the aim of the present study is to investigate the performance of latter one in alleviating Al injury. To perform the study objectives, long period pretreatment with Ca (without Al) was done which ensures the high uptake of this element into the plant tissues. That is, a sufficient amount of Ca accumulation by the plant was accomplished before Al treatment. A companion study was also conducted to investigate the effects of supply of 2500 *μ*M Ca during, before, and after Al treatment on root growth. This study will also clarify the importance of accumulated Ca in the root tissues during, before, and after Al treatment in alleviating Al injury.

## 2. Materials and Methods

### 2.1. Plant Materials and Growth Conditions

A Bangladeshi wheat (*Triticum aestivum *L.) cultivar, Kalyansona, was used in our research work. Seeds were sterilized with 1% NaClO, stirred for 10 min, washed in deionized water, and soaked in distilled water for 24 h. Then the seeds were placed on planting trays with nylon fabric screen for germination in a plastic box containing full nutrient solution (mgL^−1^): 50N (as NH_4_NO_3_), 20P (Na_2_HPO_4_·12H_2_O), 50K (K_2_SO_4_), 3Fe (FeSO_4_·7H_2_O), 0.5Mn (MnSO_4_·4H_2_O), 0.2Zn (ZnSO_4_·7H_2_O), 0.5B (H_3_BO_3_), 0.05Cu (CuSO_4_·5H_2_O), 0.05Mo {(NH_4_)_6_Mo_7_O_24_·4H_2_O}, 10Ca (CaCl_2_·2H_2_O) and 10Mg (MgCl_2_·6H_2_O), and kept for 7 d at pH 5. The seedlings by then had developed 5–7 roots. The seedlings were then transferred to 3.5 L pots containing 125, 625, and 2500 *μ*M Ca along with other nutrients. The pH of these treatment solutions was adjusted to 5.0 with NaOH and HCl, and these solutions were renewed every 3 d. Treatments consisted of three replications and each of them was composed of 3 pots. Each pot had 4 plants. A control plot was set up with 625 *μ*M Ca from the beginning. Constant aeration of the nutrient solution was provided. After two weeks, harvest one (H1) was performed with half seedlings, when growth difference appeared (observed visually) due to different levels of Ca. Then the remaining plants were exposed to 100 *μ*M Al containing only 125 *μ*M Ca at pH 4.5 for 6 d. The solutions were renewed every 3 d. Constant aeration to the nutrient solution was also provided. Then harvest two (H2) was performed at 6 d after Al treatment. At each harvest, the roots were washed in water and root lengths were measured. Then the roots were used for chemical analyses.

In the companion study, same plant materials and same nutrient solution were used. During Al treatment, other nutrients were not applied except Ca. In this study, 250 *μ*M Ca was considered as low dose and Ca treatment duration before Al treatment was reduced to 7 d instead of 14 d. Three weeks treatment duration was designated by three letters either L (250 *μ*M Ca) or H (2500 *μ*M Ca) consecutively (such as, LLL, HLL, LHL, LLH, and cLLL). C stands for the control (without Al). In the first week, plants were treated with 250 and 2500 *μ*M Ca without Al (before Al treatment). In the second week, plants were treated with 250 and 2500 *μ*M Ca with 100 *μ*M Al (during Al treatment) but the control was kept without Al. In the third week, plants were treated with 250 and 2500 *μ*M Ca without Al (recovery phase). At the end of every week, one-third plants were harvested and root lengths were measured. Here, the harvests were designated as HI, HII, and HIII.

### 2.2. Determination of Ca and Al Contents in Roots

After completion of root growth measurement, the harvested plants (root and shoot) were dried in an oven at 70–800°C for 24 h and dry weights were determined. The Al content in roots was determined as described [8]. Briefly, 20 mg dry root samples were digested using 10 N H_2_SO_4_ and 30% H_2_O_2_. Then Al in the samples was determined by pyrocatechol violet (PCV) method using a spectrometer [14]. Calcium was determined by atomic absorption spectrophotometry.

### 2.3. Extraction and Measurements of Ca and Al in the Apoplastic Fluid (AF)

The method was used as described [15]. After 6 d exposure to 100 *μ*M Al, about 2 cm (from the apex) long roots were detached from a plant of each treatment, weighed, vacuum-infiltrated with demineralized water, blotted, and reweighed. The infiltrated roots were arranged on strips (2 cm × 10 cm) of thin polyvinyl sheeting (cut-up of plastic shopping bags). The all cut ends of roots were arranged in the same direction as described [16]. The strips were rolled around to a cylindrical plastic tube with enough pressure to give a tight roll. Then the tube was placed in a 50 mL plastic syringe with 1.5 mL Eppendorf cup at its tip. The roots were centrifuged, with their cut ends pointing centrifugally at 440 g for 15 min to obtain the AF. Calcium in the AF was measured by atomic absorption spectrometry after digesting the AF with H_2_SO_4_-H_2_O_2_. Aluminum in the AF was determined by the same method as described above.

## 3. Results

### 3.1. Plant Growth

**Figure 1:**
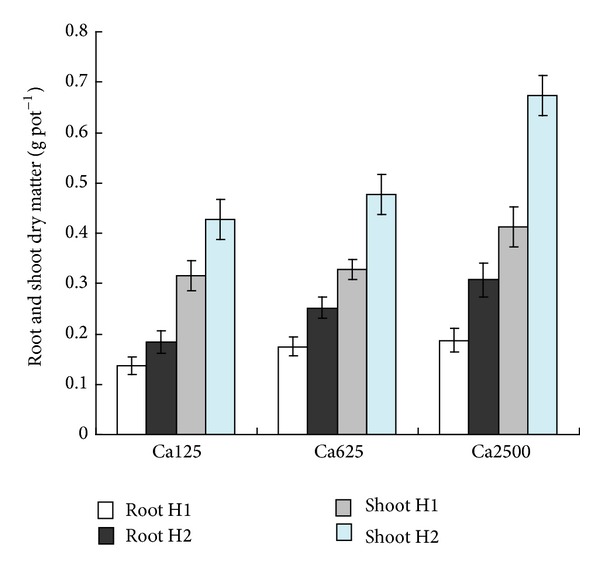
Effects of different levels of Ca supply on root and shoot dry matter of wheat seedlings without Al at H1 and with Al at H2. After 14 d of Ca treatment, the seedlings were exposed to 100 *μ*M Al for additional 6 d for H2. Values are the means ± SE of three replicates.

**Figure 2:**
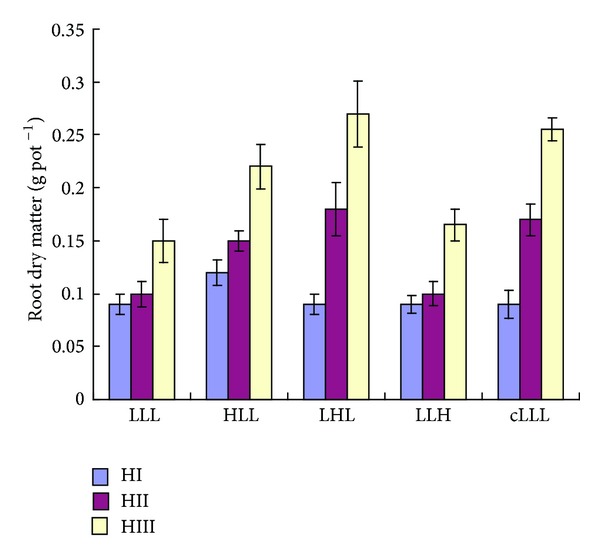
Effects of exogenous Ca supply during, before and after 100 *μ*M Al treatment on root growth in wheat plants. Three letters either L and/or H indicate three weeks and Ca level (L = 250 *μ*M, H = 2500 *μ*M). C stands for control. Values are the means ± SE of three replicates.

In the absence of Al (H1), increasing supply of Ca from 125 to 2500 *μ*M for 14 d had adistinct effect on the shoot growth as well as on root growth (Figure 1). The lowest shoot and root dry matter was recorded at 125 *μ*M Ca. Similar trend of root dry matter yields was also observed in the companion study at HI (Figure 2). That is, 7 d culture with 2500 *μ*M Ca before Al treatment (HLL) produced 20% higher root dry matter than the control with 250 *μ*M Ca (cLLL). The root dry matter in other plots was similar to the control (Figure 2).

Under Al stress conditions (H2), the plants with different Ca supply before Al treatment responded to the same level of Al in different ways (Figure 1). Dry matter production is one of the best indicators of Al toxicity. Severe Al toxicity was found in those plants treated with 125 *μ*M Ca before Al treatment. It was the highest (104%) in the control plot (data not shown). About 34, 46, and 62% increase in root dry matter was recorded at H2 in comparison with their corresponding root dry matter at H1 in the plots pretreated with 125, 625, and 2500 *μ*M Ca, respectively. The shoot dry matter production in the presence of Al was greater than the root dry matter production and showed a similar tendency.

**Figure 3:**
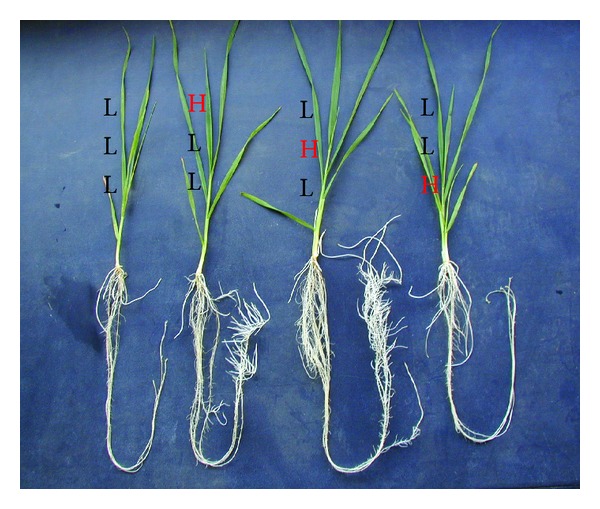
Effects of exogenous Ca supply during, before, and after 100 *μ*M Al treatment on root regrowth in wheat plants at the recovery phase (7 d treatment without Al). Three letters either L and/or H indicate three weeks and Ca level (L = 250 *μ*M, H = 2500 *μ*M).

In case of the companion study, 100 *μ*M Al was applied during the second week. After 7 d exposure to 100 *μ*M Al, root dry matter at HII was in the following order: LHL > HLL > LLH ≥ LLL (Figure 2). The results also showed that 2500 *μ*M Ca supply during Al treatment was 1.18 times more effective than the Ca supply before Al treatment in alleviating Al injury. During recovery phase (last week without Al), new root development was only observed in LHL and HLL plots (Figure 3). It was also found that new root development capacity was greater in the plants with 2500 *μ*M Ca during Al treatment (LHL) than the plants with the same level of Ca before Al treatment (HLL). Furthermore, the root tip of the former (LHL) was not strongly affected as observed in HLL. New root development at the root tips was not observed in the plants with 2500 *μ*M Ca for 7 d after Al treatment (LLH). As a consequence at HIII, about 6% more roots dry matter than the control was observed in LHL, whereas it was 88% of the control roots dry matter in HLL (Figure 2). The results indicated that high Ca supply during Al stress recovered Al injury more quickly than high Ca supply before Al stress or during recovery phase (without Al). However, there was no big difference in root dry matter in those plants with 2500 *μ*M Ca (LLH) and 250 *μ*M Ca (LLL) during the recovery phase, although more new roots were observed at the base of shoots in LLH than LLL (Figure 3).

### 3.2. Ca and Al Contents in Roots

In the absence of Al (H1), Ca content in the roots increased with increasing supply of Ca in the culture solution (Table 1). The highest Ca content was recorded in the roots with 2500 *μ*M Ca for 14 d before Al treatment. Calcium content in roots decreased drastically in the presence of Al (H2). The plants with the highest Ca (3010 *μ*gg^−1^ DM) content at H1 showed a remarkable reduction in Ca (197 *μ*gg^−1^ DM) content after 6 d exposure to 100 *μ*M Al. Aluminum was undetected in the plant at H1 and the control plant at H2. The plants pretreated with different levels of Ca had different Al content in the roots at H2. The highest Al content was determined in the roots with the lowest Ca supply before Al treatment. Aluminum content decreased gradually with increasing supply of Ca before Al treatment.

In the companion study, Ca content in the roots varied with the supply of Ca for 7 d before Al treatment (Table 2). Actually, Ca supply in LLL, LHL, LLH, and cLLL treatments was same during first week. Therefore, Ca content among the treatments was identical at HI. In the second week (during Al treatment), the lowest Ca content was observed in LLL and LLH. The Ca content in roots decreased drastically (about 90%) in the presence of Al (HII) in the plants pretreated with 2500 *μ*M Ca (HLL), whereas it was 44% in the plants that treated with 2500 *μ*M Ca (LHL) during Al exposure. In the control plants (cLLL), Ca uptake increased by 33% during second week treatment without Al. During recovery phase (HIII), Ca content increases in all treatments and the highest was in LHL. The highest Al content at HII was observed in LLL and LLH. It was 1.5 and 1.4 times higher than LHL and HLL, respectively. The results further showed that the difference in Al content between LHL and HLL was lesser extent than the difference observed in their root dry matter yields (Figure 2). Again, Al content at the recovery phase (HIII) was lower than the values observed during Al treatment (HII), although the trend was similar to the harvest two (HII).

### 3.3. Ca and Al Concentrations in the Apoplastic Fluid (AF)

Calcium concentration in the AF at 125 *μ*M Ca was larger than that of at 625 *μ*M Ca (Table 3). The highest Ca concentration in the AF was recorded at 2500 *μ*M Ca. Aluminum concentration in the AF of roots at H2 decreased with increasing supply of Ca before Al treatment. Aluminum concentration in the AF of the roots was reduced by 4 times, when these plants were pretreated with Ca supply from 125 to 2500 *μ*M.

## 4. Discussion

In the absence of Al, there were differences among the Ca levels in terms of plant (root + shoot) growth (Figures 1 and 2) and Ca nutrition (Tables 1 and 2). Under Al stress conditions (H2), root dry matter production occurred substantially but differentially among the Ca treatments in the presence of Al for 6 d (Figure 1). The differential growth may be the residual effect of accumulated Ca in the plant tissues. That is, long period pretreatment with high Ca supply before Al treatment resulted in enrichment of Ca status of the seedlings (Table 1), prevented the Al accumulation (Table 1) in root tissues, and subsequently increased dry matter production (Figure 1). Although the tested cultivar, Kalyansona, is sensitive to Al [7], cultivation with 100 *μ*M Al for 6 d caused a decrease in root elongation by only 44% at 125 *μ*M Ca while it was 56% within 48 h for 4 d old seedlings (data not shown). Therefore, other factors such as plant age may cause the difference in the short-term and long-term experiments as reported [5].

Severe Al injury was found in the plants that pretreated with 125 *μ*M Ca (Figure 1). It is assumed that all of the common binding sites (CBS) of the roots of these plants are not occupied by Ca during pretreatment period. Many of the CBS remain exposed as reported in our previous study [17]. During Al treatment, both the Ca^2+^ bounded and unbounded binding sites in the cell wall as well as plasma membrane may be occupied by Al since Al^3+^ has stronger affinity to the negative CBS than Ca^2+^. Such binding of Al^3+^ to the cell wall as well as plasma membrane caused the destruction of epidermal and cortical cells in the root tip, elongating zones, and proximal parts of the root metabolically and morphologically [18, 19]. This type of destruction of root cellsincreases total Al content in addition to the Al content accomplished by the intact root before destruction. In addition, Al caused disintegration of plasma membrane in barley root cells [20]. In our present study, the plasma membrane in some of the root cells was destroyed due to the attack of 100 *μ*M Al for 6 d in the presence of only 125 *μ*M Ca, and Al in these cells was possibly extracted during extraction of AF. Therefore, Al concentration in the AF may be affected. Calcium concentration in the AF at 125 *μ*M Ca was larger than that of at 625 *μ*M Ca (Table 3). Severe attack of Al was accomplished in the former (Figure 1) which possibly caused the destruction of plasma membrane of many/more cells than the latter and consequently affected apoplastic Ca concentration. The highest supply of Ca (2500 *μ*M) before Al treatmentnot only enable the Ca^2+^ ions to occupy most of the CBS of the roots [21] but also enable them present in the highest concentration in the apoplast (Table 3). According to toxicant-ameliorant competition for CBS [4], bounded Ca along with large amount of unbounded Ca in the apoplast may protect the metabolical and morphological disruption of cell wall and plasma membrane [17] in the presence of Al. This protection effect is clearly reflected on the results of low content of Al in the roots (Table 1) as well as low concentration of Al in the AF (Table 3) and increased root dry matter production (Figure 1).

The disturbance of Ca homeostasis via Ca displacement from CBS and increased cytosolic Ca level by Al is a well-established event during Al-Ca interactions in plants [20]. In our present study, the plants with high Ca supply showed a remarkable reduction in the Ca content in roots after 6 d exposure to Al (Table 1). This remarkable reduction in the Ca content may be caused not only by displacing Ca from the CBS as observed in the previous studies [4, 8] but also by the expense of Ca in the cells for continuation of root growth during Al stress (Figure 1). Shoot dry matter was also increased after Al treatment (Figure 1). The results can be explained by the expense of both shoot containing Ca and translocated Ca from roots since Al decreased Ca content in the roots (Table 1).

In the companion study, the highest root growth at HII (Figure 2) as well as the highest Ca content (Table 2) was observed when 2500 *μ*M Ca was supplied during Al treatment. The results also showed that 2500 *μ*M Ca supply before Al treatment is able to alleviate Al injury but lesser extent than the same level Ca supply during Al treatment (Figure 2). The difference of Ca content between LHL and HLL (Table 2) showed the similar trend to the difference of root dry matter yields (Figure 2). Therefore, it can be speculated that accumulated Ca of the wheat plants plays an important role to alleviate Al injury (expressed by root dry matter yields) during Al treatment. But 2500 *μ*M Ca supply (LLH) at the recovery phase could not improve root growth inhibition caused by Al toxicity (Figure 2), and new root development at the root tips was not observed in this plot (Figure 3). This may be the result of destruction of the roots tips having meristematic tissues during Al treatment. However, new root initiation at the base of shoots in LLH (Figure 3) indicates the recovery from Al injury due to high Ca supply in culture solution. The plants in LLH may recover much faster from Al injury than those in LLL. In conclusion, pretreatment with high doses of calcium on the injury induced by aluminum in wheat plants, especially in roots, may be beneficial to protect against subsequent aggression by aluminum. Although the interaction between Ca and Al has been already extensively studied, the potential benefit of Ca pretreatment may be of interest to the field.

## Figures and Tables

**Table 1:** Calcium and Al contents (*μ*gg^−1^ dry matter) in the roots obtained before (H1) and after (H2) exposure to 100 *μ*M Al for 6 d. Values are the means ± SE of three replicates.

Treatments	Ca content		Al content	
	H1	H2	H1	H2
Control	1885 ± 355	2375 ± 445	ND	ND
Ca 125	980 ± 122	95 ± 12	ND	5791 ± 825
Ca 625	1885 ± 355	112 ± 15	ND	5025 ± 625
Ca 2500	3010 ± 585	197 ± 31	ND	3883 ± 337

ND (not detected).

**Table 2:** Calcium and Al contents (*μ*gg^−1^ dry matter) in the roots obtained before (HI), during (HII), and after (HIII) exposure to 100 *μ*M Al for 7 d. Values are the means ± SE of three replicates.

#	HI		HII		HIII	
	Ca	Al	Ca	Al	Ca	Al
LLL	927 ± 35	ND	127 ± 16	5134 ± 387	159 ± 22	4325 ± 503
HLL	2915 ± 275	ND	305 ± 22	3883 ± 122	387 ± 51	2552 ± 202
LHL	927 ± 27	ND	515 ± 31	3415 ± 272	649 ± 105	2347 ± 131
LLH	935 ± 25	ND	132 ± 12	5101 ± 402	193 ± 21	4061 ± 263
cLLL	942 ± 23	ND	1245 ± 98	ND	1727 ± 235	ND

^#^Treatment condition: 3 letters indicate 3 weeks. c stands for control. L = 250 *μ*M Ca.

H = 2500 *μ*M Ca, and ND: not detected.

**Table 3:** Calcium and Al concentrations in the apoplastic fluid of the roots treated with 100 *μ*M Al for 6 d (H2). The wheat seedlings were pretreated with different levels of Ca for 14 d before Al treatment. Values are means ± SE of three replicates.

Treatments (*μ*M)	Concentrations *μ*g mL^−1^ AF	
	Ca	Al
Ca 125	6.5 ± 0.28	1.15 ± 0.10
Ca 625	5.0 ± 0.57	0.35 ± 0.07
Ca 2500	12.5 ± 0.50	0.28 ± 0.05
